# The effect of leg compression garments on the mechanical characteristics and performance of single-leg hopping in healthy male volunteers

**DOI:** 10.1186/s13102-015-0005-x

**Published:** 2015-04-19

**Authors:** Amitabh Gupta, Joshua John Bryers, Peter James Clothier

**Affiliations:** School of Science & Health, University of Western Sydney, Narellan Road, Campbelltown, Australia

**Keywords:** Fatigue, Vertical stiffness

## Abstract

**Background:**

Compression garments (CG) are commonly used by athletes to improve motor performance and recovery during or following exercise. Numerous studies have investigated the effect of CG on physiological and physical parameters with variable results as to their efficacy. A possible effect of commercially available CG may be to induce a change in leg mechanical characteristics during repetitive tasks to fatigue. This investigation determined the effect of CG on performance and vertical stiffness during single-leg-hopping to exhaustion.

**Methods:**

Thirty-eight healthy, male participants, mean (SD) 22.1 (2.8) years of age performed single-leg hopping at 2.2 Hz to volitional exhaustion with a CG, without CG and with a sham. Differences in total duration of hopping (1-way repeated ANOVA) and dependant variables for the start and end periods (2-way repeated ANOVA) including duration of flight (t_f_), loading (t_l_) and contact (t_c_) phases, vertical height displacement during flight (z_f_) and loading (z_l_) phases, normalised peak vertical ground reaction force (F_zN_) and normalised vertical stiffness (*k*_N_), were determined. Bonferroni correction was performed to reduce the risk of type 1 error.

**Results:**

There was no significant difference (p = 0.73) in the total duration of hopping between conditions (CG (mean (SD)) 89.6 (36.3) s; without CG 88.5 (27.5) s; sham 91.3 (27.7) s). There were no significant differences between conditions for spatiotemporal or kinetic characteristics (p > 0.05). From the start to the end periods there was no significant difference in t_l_ (p = 0.15), significant decrease in t_f_ (p < 0.001), z_f_ and z_l_ (p < 0.001) and increase in t_c_ (p < 0.001). There was also a significant increase in *k*_N_ from start to end periods (p < 0.01) ranging from 9.6 to 14.2%.

**Conclusions:**

This study demonstrates that commercially available CG did not induce a change in spatiotemporal or vertical stiffness during a fatiguing task. The finding that vertical stiffness increased towards the end of the task, while hopping frequency and duration of loading were maintained, may indicate that there was an alteration to the motor control strategy as fatigue approached.

**Trial registration:**

Current Controlled Trials ACTRN12615000240549. Registered 17 March 2015.

## Background

Compression garments (CG) are used within the medical community to treat conditions such as chronic venous insufficiency [1,2], burns and scarring [3]. More recently recreational and elite level athletes have used CG in an effort to improve performance [4,5]. Numerous investigations have aimed to determine the physiological effects that CG have on the body during and after physical exercise [6] with relatively few studies investigating the possible effects on mechanical characteristics during athletic activity.

Greater mean power output was demonstrated for volleyball players wearing an above knee CG during 10 repeated vertical jump efforts [7]. Furthermore, improvements in endurance running were observed while wearing calf compression stockings, however, these were not attributable to the small improvement in aerobic capacity that was also observed [8]. The mechanical effect of wearing above knee CG was demonstrated with decreased muscle oscillation in the sagittal plane during a countermovement jump test [9]. Doan et al. also reported greater squat depth during the countermovement jump test in men and a trend towards reduced hip flexion during a 60 m sprint test in both sexes. The ability of CG to create a mechanical effect by compressing the thigh has been shown to affect both muscle oscillation and joint range of motion using either fitted or undersized compression garments [7]. These findings suggest that a change in motor performance could be induced due to changes in both the joints and muscles underlying the CG as suggested in a recent review [10].

The effect of CG on motor performance parameters and leg mechanical characteristics during rapid and repeated loading of the limb to exhaustion was of specific interest in this study. This was because CG are widely used by athletes training and competing in field sports and athletics, all of which require repeated loading of the lower limb over prolonged periods and often to exhaustion. To mimic the repeated effort of the stretch-shorten-cycle during a rapid loading task, the effect of CG whilst hopping was determined. Hopping has been shown to have relatively lower variability in performance characteristics than running [11-13]. Therefore, it was deemed a task that may be sensitive enough to detect the possible effect of CG on leg mechanical characteristics. The purpose of this investigation was to determine the effect of CG on spatiotemporal and leg mechanical characteristics during single-leg hopping to volitional exhaustion.

## Methods

### Participants

Thirty-eight healthy, recreationally active males with a mean (SD) age of 22.1 (2.8) years, 181.0 (6.7) cm in height and 78.3 (10.8) kg in body mass were recruited from the student population at the institution where the study was conducted. Participants were classified as recreationally active if they participated in sport or exercise for between 3–15 hours per week for the 6 months prior to testing and excluded if they were classified as sedentary (<3 hours/week) or high performing (>15 hours/week). Participants were excluded if they reported pain or injury of the lower limb or spine on the day of testing or in the 6 months prior to testing. Ethical approval was obtained from the University of Western Sydney Human Research Ethics Committee (H9066). All participants provided written and informed consent prior to the commencement of testing and were informed that they could withdraw from the study at any time during the testing procedure.

### Testing procedure

All hopping trials were performed barefoot on the self-reported, preferred hopping leg [11,14] on a single day. Each participant completed a familiarisation period followed by a series of single-leg hopping efforts on a force plate (Kistler, model 9286B, Switzerland) in synchrony with a metronome (freeware: http://www.nch.com.au/metronome/) that emitted an audible tone at 132 beats/minute (2.2 Hz) from a personal laptop (Dell, Studio 1555 - PP39L, USA). Temporal and vertical ground reaction force (F_z_) data were collected at a sampling frequency of 1000 Hz during trials via an A/D convertor (Kistler Type 5691A1) and recorded on a personal laptop (Lenovo, W700, USA) using Bioware® software (Version 4.0, Type 2812D).

Instructions to each participant on how to perform each trial were; to stop hopping when he felt exhausted, could not hop in synchrony with the metronome and to avoid contacting the heel with the force plate. Participants were required to place their hands on their hips for the duration of each trial whilst focusing on a piece of tape placed on the ground 1.2 m in front of the force plate. A warm-up was performed by each participant and involved walking at a self-selected pace for 5 minutes and performing stretches of the triceps surae, muscle and hamstring muscle groups [15]. The familiarisation period involved hopping in synchrony with the metronome aiming to land on the sound of the audible tone while maintaining a position close to the centre of the force plate avoiding horizontal translation. Each effort was limited to 10 s with a minimum 60 s rest between efforts to reduce the risk of fatigue. Once the primary investigator was satisfied that the participant could perform the hopping task as instructed, testing commenced.

The order of testing of the three hopping conditions (garment, control and sham) was randomly determined by asking each participant to choose a marked card that was placed face down. A 10 min rest period was maintained between each hopping effort to ensure that the participant had adequately recovered before commencing the next trial [16-19]. The CG was a full length garment (Skins™) from the pelvis to the ankle. The size of the garment that each participant used during testing was based on the manufacturer’s guidelines (http://www.skins.net/en-AU/why-skins/unique-sizing-guide.aspx) consistent with the literature [7].

Hopping without a CG and with a sham intervention required the participant to wear loose fitting, above knee shorts. The sham hopping condition also involved applying a 200 mm length of 50 mm wide rigid sports tape (Leuko™ sports tape) over the knee extensor and ankle plantarflexor muscle groups. The midpoint of the thigh and posterior shank were determined and marked by measuring the length from the anterior superior iliac crest to the superior border of the patella and from the popliteal fossa to the floor, respectively. Sports tape was applied vertically, without stretch, over the midpoints. Participants were informed that the tape aimed to provide sensory feedback via application to the skin and that this may assist in the ability of the muscle to perform repeated contractions required to complete the hopping task. A thorough literature search did not find any evidence to suggest that rigid sports tape applied along the line of pull of the quadriceps femoris and triceps surae muscles would affect motor performance during repeated submaximal single-leg hopping and was considered an appropriate sham intervention as it was not applied over a joint to limit joint motion.

The primary investigator provided verbal instructions during the trials to reinforce the criteria on how to perform each hopping condition. Following the three trials, participants performed a warm-down (walking or jogging at a self-selected pace on a treadmill) and a series of static lower limb muscle stretching exercises [20,21].

### Data processing

Vertical ground reaction force (F_z_) data for each trial were filtered using a Butterworth filter with a low-pass cut-off of 33 Hz (Bioware version 4.0, Type 2812D). Data were exported to an excel spread sheet (Microsoft Office Excel, 2007) for further analysis. A hopping cycle was defined as a consecutive flight phase and contact phase from the F_z_ recording [22].

Hop cycles included in the analyses had to be ±10% of the target hopping frequency of 2.2 Hz, ranging from 1.98 to 2.42 Hz. All hop cycles for all trials were found to be ±10% of 2.2 Hz. This allowed a valid comparison between conditions and start and end periods, as it has been previously determined that leg mechanical characteristics may change dependant on the hopping frequency [23]. The dependant variables for each hop cycle during the start (first 10 consecutive hop cycles) and end (last 10 consecutive hop cycles) periods were determined. A mean score for each dependant variable was calculated for the start and end periods for each trial.

The total duration of hopping, from the start of the first hop cycle during the start period to the end of the contact phase of the last hop cycle during the end period, was determined for each trial. For each hop cycle the following dependant variables were determined: duration of flight phase (t_f_) (from start of flight to initial contact (IC)), duration in loading phase (from IC to peak F_z_) and duration of contact phase (from IC to toe-off), normalised peak F_zN_ (peak F_z_ (N)/BM (kg)), vertical displacement of the centre of mass (COM) during loading (z_l_) and normalised vertical leg stiffness (*k*_N_).

Initial contact and toe-off for each hop cycle were labelled as the first and last F_z_ value ≥10 N respectively during the contact phase. Peak F_z_ was the greatest value during the contact phase and defined the end of the loading phase. Total hopping duration for each trial was calculated as the elapsed time between the commencement of the flight phase for the first hop cycle of the start period to toe-off for the final hop cycle in the end period. The duration of a single hop cycle was from commencement of flight phase to toe-off.

To determine normalised vertical stiffness (*k*_N_), the quotient of normalised F_z_ (normalised to body mass (kg)) and vertical displacement of the COM during the loading phase (z_l_) equations that represent the Law of Falling bodies (equations 1, 2 and 3) were used.

1 $$ {z}_f=\frac{1}{2}\cdot g\cdot {\left(\frac{t_f}{2}\right)}^2 $$

Where z_f_ was the vertical displacement of the centre of mass (COM) from peak height during flight phase to initial contact (IC), g was the acceleration due to gravity (9.81 m.s^−1^) and t_f_ was the duration of flight phase. This method assumes that the velocity of the COM at the peak height during flight phase was 0 m.s^−1^ as there was a change in direction of the COM that occurred at half flight phase.

The velocity at IC was then determined by the following equation

2 $$ {\mathrm{v}}_{\mathrm{i}}=\sqrt{2.g.{z}_f} $$

Where v_i_ was the velocity at IC, g was the acceleration due to gravity (9.81 m.s^−2^) and z_f_ was vertical displacement of the COM during the second half of flight phase.~

Vertical displacement of the COM during loading phase (z_l_) from IC to peak F_z_ was then calculated using the following equation

3 $$ {z}_l=\frac{1}{2}\left({v}_i+{v}_f\right)\cdot {t}_l $$

Where z_l_ was the vertical displacement of the COM during loading phase, v_i_ was the velocity at IC, v_f_ was velocity at peak vertical ground reaction force (F_z_) and assumed to be 0 m.s^−1^ and t_l_ was the duration of loading phase.

### Statistical analyses

A one-way repeated measures analysis of variance (ANOVA) was performed to determine differences between trials for total hopping duration. A two-way repeated measures ANOVA was performed to determine differences in spatiotemporal characteristics and vertical stiffness between hopping conditions and the start and end periods. Mauchly’s test of sphericity was utilised to assess the null hypothesis that the error covariance matrix of the orthonormalized transformed dependant variables was proportional to an identity matrix. If this was found to be statistically significant (p < 0.05) then the degrees of freedom was adjusted using the Greenhouse-Geisser epsilon to determine the within subjects effects. Statistical significance was accepted at p < 0.05 with Bonferroni correction made for all analyses to reduce the risk of making a type 1 error.

## Results

There was no significant difference in the total duration of hopping between hopping with a CG (mean (SD)) 89.6 (36.3) s, without a CG 88.5 (27.5) s and the sham intervention 91.3 (27.7) s. There was no significant difference in t_f_ between hopping conditions with a significant decrease in t_f_ from start to end periods (p < 0.001) (Table 1). There was no significant difference in t_l_ between hopping conditions or between start and end periods (Table 1). There was no significant difference in t_c_ between hopping conditions (p = 0.06) and a significant increase in t_c_ from the start to end periods (p < 0.001) (Table 1).

**Table 1:** **Spatiotemporal and leg mechanical characteristics (mean (SD))**

	Garment		No Garment		Sham	
	Start	End	Start	End	Start	End
Flight phase (ms)*	115 (24)	103 (231)	115 (22)	99 (24)	111 (22)	100 (24)
Loading phase (ms)	166 (16)	167 (18)	165 (14)	170 (22)	168 (17)	171 (18)
Contact phase (ms)*	339 (24)	351 (25)	340 (22)	355 (27)	344 (25)	355 (26)
z_f_ (mm)*	17 (7)	14 (6)	17 (7)	13 (6)	16 (6)	13 (7)
z_l_ (mm)*	46 (7)	42 (8)	46 (8)	41 (7)	45 (7)	41 (8)
F_zN_ (N.kg^−1^)	24.0 (2.6)	23.8 (2.8)	24.2 (2.7)	23.5 (3.0)	23.9 (2.4)	23.3 (2.7)
*k*_N_ (N.kg^−1^.m^−1^)*	539.4 (67.0)	607.6 (135.7)	544.1 (74.2)	611.8 (123.4)	550.0 (86.8)	595.1 (101.4)

z_f_ - vertical displacement of the COM during flight phase; z_l_ - vertical displacement of the COM during loading phase; F_zN_ - Normalised Peak Vertical Ground Reaction Force (N.kg^−1^); *k*_N_ - Normalised Vertical Leg Stiffness; *Statistically significant difference between start and end periods for each hopping condition (p < 0.01).

There was no significant difference in z_f_ between hopping conditions with a significant decrease in z_f_ from start to end period (p < 0.001) (Table 1). There was no significant difference in z_l_ between hopping conditions with a significant decrease in z_l_ from start to end periods (p < 0.001) (Table 1). There was no significant difference in F_zN_ between hopping conditions or between start and end periods (Table 1). There was no significant difference in *k*_N_ between hopping conditions with a significant increase in *k*_N_ from start to end periods (p < 0.01) ranging from a 9.6 to 14.2% (Table 1).

## Discussion

This investigation determined that CG did not have any significant effect on spatiotemporal characteristics or vertical stiffness during single-leg hopping to exhaustion. This investigation determined that there was a significant increase in *k*_N_ as participants approached volitional exhaustion during single-leg hopping. Previously it has been demonstrated that during double-leg hopping to fatigue, ground contact time increased while maintaining a hopping frequency of 2 Hz (±2.5%) [24], supporting the finding of the current study that also demonstrated an increase in t_c_ and a decrease in t_f_ at the end compared to the start period while hopping at 2.2 Hz.

The current findings suggest that CG did not influence lower limb mechanical characteristics when the leg was modelled as a massless spring and *k*_N_ was calculated as the quotient of F_z_ and z_l_. However, lower limb geometry and the muscle activation characteristics that determine the leg length, may change during the task as demonstrated during hopping [25-27]. Changes to neuromuscular properties have also been reported with the finding of invariant medial gastrocnemius muscle function and change in the synergy between knee and ankle joint moments [28]. The current study found that there were changes from the start to end periods of hopping in spatiotemporal characteristics and vertical stiffness that would have most likely have been due to changes in neuromuscular and kinematic adaptations. However, there was no influence of CG on performance as described by spatiotemporal characteristics and vertical stiffness during a repeated submaximal task to volitional exhaustion, even though previous literature has reported alterations to hip joint ROM using CG [19].

Vertical stiffness values in the current study were similar to previously published literature [14,18,29-34]. An increase in *k* has been observed when hopping frequency increased [31,35,36], participants hopped to a greater height [37], contact time decreased [26] and hopping surface stiffness decreased [38]. In contrast, *k* remained unchanged when assessed during double-leg hopping, following a bout of repeated squatting at submaximal loads (30% of body weight) [14]. Although the current study primarily induced fatigue of the ankle plantarflexor muscle group which has been shown to modulate the performance of hopping [37-39], the findings contrasted those of Padua et al. who induced fatigue in the quadriceps femoris muscle group utilising a squatting exercise. Therefore, it is plausible that adaptation of vertical leg stiffness is sensitive to the changes at a specific joint [40] or muscle group.

The findings of reduced vertical muscle oscillation and reduced error, measured for hip flexion while wearing above knee CG, are suggested mechanisms that effected a lower decline in mean power output of 10 repeated vertical jumps, following different fatiguing protocols [19]. The current study demonstrated that whole leg CG did not affect the increase in *k*_N_ observed at the end of single-leg hopping to exhaustion. Although changes to muscle oscillation and proprioception have previously been shown, leg mechanical characteristics represented by vertical stiffness were not affected with the use of CG. More importantly, CG did not affect the total duration of hopping or spatiotemporal characteristics that describe the motor performance of single-leg hopping. These findings are consistent with a previous investigation that demonstrated no change in peak torque or total work performed following repetitive, high intensity isokinetic knee flexion/extension [41]. Full leg Skins™ CG were used in the current study and extended from the pelvis to above the ankle, covering the hip and knee but not the ankle joint. The CG may restrict motion at the hip and knee joints and affect joint function due to compression of the muscles that act about the hip, knee and ankle. It is possible that there may in fact have been changes affecting a single joint or specific group of muscle only; however, the measure of vertical stiffness assumes the leg to act as a spring and may not be sensitive enough to detect changes at a joint or muscle level due to interaction between segments [42,43]. Although there may be evidence of CG reducing muscle oscillation [19], CG have not yet shown to lead to a change in leg mechanical characteristics during repetitive lower leg movements as during functional activities. This may be due to the method of assessment which may not be sensitive enough to detect the alterations in joint or leg mechanical characteristics when wearing CG. An individually customised CG has the potential to provide either greater or more uniform compression along the limb and may induce changes in mechanical characteristics. Furthermore, it may be possible that the underlying effect of CG is on blood flow, muscle temperature or sensory input rather than mechanical characteristics.

It has been recommended to determine the pressure applied by the CG to the soft tissues as this may likely affect the physical and physiological responses [10]. The current study aimed to determine the effect of CG most commonly worn as a prefabricated garment in commercially available sizes. The varieties of garment sizes are based on generic anthropometric features such as height, mass and limb circumference [10]. Therefore, as an intervention, the current study aimed to only determine the possible effects of whether a commercially available garment, when fitted to the manufacturer’s guidelines, affected physical performance. This study did not aim to investigate the effect of CG due to the degree of compression provided by CG. It follows that CG may have had an effect on muscle activation characteristics and future studies should aim to assess the possible effect of CG on muscle activity during fatiguing tasks.

## Conclusions

The aim of this study was to determine the effect of CG on performance and leg mechanical characteristics during single-leg hopping to exhaustion. The findings were that commercially available CG did not induce a change in spatiotemporal or vertical stiffness during a fatiguing task. However, there was an increase in vertical stiffness towards the end of the task, while hopping frequency and duration of loading were maintained. These findings may indicate that there was an alteration to the motor control strategy as fatigue approached. It is plausible that individually customised CG may induce alterations in performance or leg mechanical characteristics or that the previously reported benefits of CG are due to other physiological mechanisms.
